# The prognostic potential of an immunology score in patients treated with curative surgery for non-small cell lung cancer

**DOI:** 10.1007/s00432-026-06562-1

**Published:** 2026-07-21

**Authors:** Sara Witting Christensen Wen, Signe Timm, Morten Borg, Ole Hilberg, Torben Frøstrup Hansen, Christa Haugaard Nyhus, Torben Schjødt Hansen, Line Nederby

**Affiliations:** 1 Department of Biochemistry and Immunology, Lillebaelt Hospital Vejle, University Hospital of Southern Denmark, Beriderbakken 4, 7100 Vejle, Denmark; 2 Department of Regional Health Research, Faculty of Health Sciences, University of Southern Denmark, Campusvej 55, 5230 Odense, Denmark; 3 Department of Oncology, Lillebaelt Hospital Vejle, University Hospital of Southern Denmark, Beriderbakken 4, 7100 Vejle, Denmark; 4 Department of Medicine, Lillebaelt Hospital Vejle, University Hospital of Southern Denmark, Beriderbakken 4, 7100 Vejle, Denmark

**Keywords:** Non-small cell lung cancer, Lung cancer, Surgery, Biomarkers, Immunology, Inflammation

## Abstract

**Purpose:**

Curative surgery is the best treatment for patients with early-stage non-small cell lung cancer (NSCLC), but around 40% experience recurrence impacting survival. Does the individual immunological status affect the prognosis? This study investigated four immunology markers in relation to recurrence and survival after curative surgery for NSCLC.

**Methods:**

Natural Killer cell activity (NKA), C-reactive protein (CRP), Interleukin 6 (IL6) and Transforming growth factor beta (TGFβ) were analysed before surgery and at three months after surgery for NSCLC.

**Results:**

Of the 103 enrolled patients, 43 experienced recurrence over a period of median 71 months. The biomarkers were unsuitable for detecting recurrence with area under the receiver operating characteristics curve ranging from 0.52 to 0.58. Baseline CRP and TGFβ were significantly associated with overall survival (OS), and a score based on all four biomarkers demonstrated statistically significant prognostic impact on both recurrence free survival (RFS) and OS. The biomarker score was an independent prognostic factor when adjusting for stage and surgery type, hazard ratio 3.18 (95% CI 1.53–6.61, *p* = 0.002) for OS with borderline significance for RFS, hazard ratio 1.93 (95% CI 1.06–3.51, *p* = 0.032).

**Conclusion:**

Individually, NKA, CRP, IL6 and TGFβ were not suited for predicting disease recurrence after curative surgery for NSCLC. However, the combined biomarker score was significantly associated with OS. The score may give an indication of the immunological status of the patient, which could be useful for pre- or post-surgery assessment, in relation to immunotherapy or other oncologic treatments if future studies can prove clinical utility.

**Supplementary Information:**

The online version contains supplementary material available at https://doi.org/10.1007/s00432-026-06562-1.

## Introduction

Patients with lung cancer have a poor prognosis with an estimated 5-year survival of 25% in Denmark (Christensen et al. 2024; Rasmussen and Jakobsen 2025). Curative surgery is the best treatment option for early-stage patients with non-small cell lung cancer (NSCLC) improving the 5-year survival to 65% in recent years in Denmark (Rasmussen and Jakobsen 2025; Zer et al. 2025). However, many different aspects of the patient’s overall health can impact their survival making it difficult to predict the individual prognosis.

The immune system is known to play an important role in tumour development and metastatic potential (Hanahan and Weinberg 2011). A dysregulated immune system can be tumour-promoting, and the tumour can create a micro-environment which favours rapid growth and invasion (Hanahan and Weinberg 2011; Hanahan 2022). A pro-inflammatory immune profile can provide the tumour cells with growth factors to help them sustain a proliferative state thereby fostering tumour progression (Grivennikov et al. 2010; Rahal et al. 2024). Regulatory immune cells such as regulatory T lymphocytes and myeloid-derived suppressor cells can also be recruited by the tumour to help it avoid destruction by cytotoxic T lymphocytes and natural killer (NK) cells (Jeong et al. 2025).

The impact of the immune system has been suggested in all stages of tumour development and may be especially important after curative treatment, where the immune system may be able to clear any remaining abnormal cells. On the contrary, an abnormal immune response may favour seeding and survival of tumour cells in a distant location giving rise to a metastasis. A perioperative pro-inflammatory cytokine profile has been associated with increased risk of recurrence in patients with NSCLC undergoing curative surgery (De La Fuente et al. 2025) and shorter overall survival (OS) in patients with NSCLC across multiple stages (Wang et al. 2024).

C-reactive protein (CRP) is one of the most widely used markers of systemic inflammation. It is non-specific and is known to be increased in cancer patients as well as during infection (Black et al. 2004; Allin and Nordestgaard 2011; Muller et al. 2019). CRP is mainly produced in the liver but can also be produced in other cells in response to interleukin (IL) 6 (Black et al. 2004; Allin and Nordestgaard 2011). Increased levels of IL6 have been associated with a poor prognosis in NSCLC (Liu et al. 2022; Zhang et al. 2024). IL6 is one of the most well-known activators of the signal transducer and activator of transcription factor 3 (STAT3) pathway, which is crucial in regulating cell proliferation and angiogenesis (Liu et al. 2014; Tanaka et al. 2014; Hu et al. 2024).

NK cells constitute an important part of the innate immune system with their ability to destroy abnormal cells such as cancer cells (Bryceson et al. 2011; Krabbendam et al. 2021). A high number of tumour-infiltrating NK cells have been associated with a better prognosis in various solid tumours (Soo et al. 2018; Federico et al. 2022), and a high level of NK cell activity (NKA) has indicated better progression free survival (RFS) and OS in patients with NSCLC treated with immunotherapy (Choi et al. 2020; Wen et al. 2023). A low level of NKA may be caused by high levels of the cytokine transforming growth factor beta (TGFβ), which can have both pro-tumour and anti-tumour effects depending on the cellular context (Massagué 2008; Ikushima and Miyazono 2010). TGFβ has also been associated with cancer progression and resistance to immunotherapy (Derynck et al. 2021).

The present study aimed to evaluate the four inflammatory and immunologic biomarkers NKA, IL6, CRP and TGFβ before and after curative surgery for NSCLC and examine their association with recurrence and survival.

## Materials and methods

### Study participants

The patients were prospectively enrolled at the Department of Medicine, Lillebaelt Hospital Vejle, Denmark between October 2017 and February 2021. The protocol was approved by the Regional Committee on Health Research Ethics for Southern Denmark (August 25, 2017, ID S-20170100 and April 3, 2024, ID 109820). The work was carried out in accordance with the Declaration of Helsinki, and informed consent was obtained before any study related procedure. Data handling was in accordance with Danish legislation and observed the General Data Protection Regulation (EU) 2016/679.

Patients eligible for surgery with curative intent for NSCLC were consecutively recruited at the fast-track outpatient clinic at the Department of Medicine. Patients were excluded if they did not undergo surgery for any reason or if they were converted to treatment with palliative intent.

### Treatment and follow-up

Participation in the study did not influence treatment or follow-up. All patients were discussed by a multidisciplinary tumour board, where they were staged according to clinical tumour, node, metastasis (TNM) 8th edition (cTNM) (Goldstraw et al. 2016) and referred for surgery. The operation was performed at the Department of Thoracic Surgery, Odense University Hospital. Radicality and pathology TNM (pTNM) were assessed by the Department of Pathology, Odense University Hospital, based on the surgical specimen.

The patients were offered adjuvant chemotherapy based on the pTNM stage and followed at the Department of Oncology, Lillebaelt Hospital Vejle, with CT-scans of chest and abdomen at three-month intervals for the first two years and subsequently at six-month intervals for the final three years.

### Definition of endpoints

The date of disease recurrence was defined as the date on which the biopsy was performed to confirm the diagnosis. RFS was defined as the time from surgery until recurrence or death, and OS was defined as the time from surgery to death. Patients were censored at the end of follow-up on May 23, 2025, if they had not experienced recurrence or death.

### Sample collection and processing

Blood samples were collected before surgery and at the first two follow-up visits. Venipuncture was performed according to institutional guidelines and 10 mL blood was collected in a Clot Activator Tube (BD, Franklin Lakes, NJ, USA). The sample was centrifuged at 2,000xg for 10 min between 30 min and 4 h after sampling, the serum was collected into cryo vials and stored at − 80 °C until analysis. Storage time ranged from 48 to 91 months.

Analysis of NKA required 1 mL venous blood collected directly into a Promoca-containing NK Vue^®^ tube (NKMAX, Seongnam-si, South Korea). The tube was placed in an incubator at 37 °C within 15 min of sampling, and plasma was collected after 20–24 h of incubation and stored at − 80 °C until analysis. Maximum storage time was 1 month.

### Natural Killer cell activity

Estimation of NKA using the NK Vue^®^ kit (NKMAX, Seongnam-si, South Korea) has previously been reported (Nederby et al. 2018; Wen et al. 2023). Briefly, NKA was analysed using the NK Vue^®^ enzyme-linked immunosorbent assay (ELISA) (NKMAX, Seongnam-si, South Korea) according to the manufacturer’s instructions. The ELISA measures the Interferon gamma (IFNγ) secreted by the stimulated NK cells as a surrogate for NKA. The range of the ELISA assay was 62.5–2,000 pg/mL, and samples above the upper limit were re-analysed in a 1:10 dilution. In addition to kit controls, three serum controls were included (200 pg/mL, 1,500 pg/mL and 3,600 pg/mL).

### Interleukin 6

IL6 in plasma was analysed on the COBAS e801 system (Roche Diagnostics, Rotkreuz, Switzerland) using an electro chemi-luminescent immune assay principle. The lower limit of detection was 1.5 ng/L, the lower limit of quantification was 3.5 ng/L and the upper limit of quantification was 5,000 ng/L with the possibility of re-analysing the samples in a 1:10 dilution. The samples were analysed in parallel with the hospital’s routine workflow using instrumentation operating 24/7. IL-6 CalSet and PreciControl Multimarker were used for calibration and quality control (Roche Diagnostics). As stated in the kit insert, concentrations < 7 ng/L were classified as normal.

### C-reactive protein

CRP in plasma was measured alongside routine hospital analyses on the COBAS C702 system (Roche Diagnostics, Rotkreuz, Switzerland), a 24/7 automated platform, using a particle-enhanced immunoturbidimetric analysis principle. Calibration was performed using Calibrator f.a.s. Proteins (Roche Diagnostics) and both Autonorm™ Clin Chem Liq L-2 and Seronorm™ Immunoprotein Liq L-2 (SERO, Billingstad, Norway) were used as controls. The lower and upper detection limits were 0.3 mg/L and 350 mg/L, respectively. Samples with higher levels of CRP were diluted and re-analysed with an upper limit of 700 mg/L. CRP < 5 mg/L was considered normal.

### Transforming growth factor beta

TGFβ was analysed using the Human TGF-beta 1 DuoSet ELISA and the Duoset Ancillary Reagent Kit (R&D Systems, Abingdon, UK). The analyses were performed with minimal modifications as per the manufacturer’s instructions. In brief, the 96-well plates for all samples in the study were coated with capture antibody overnight. Subsequently, plates were washed and blocked in Block Buffer. Sealed plates were kept at 5 °C for up to 3 weeks. 40 µL serum was mixed with 20 µL of 1 N HCl, followed by neutralization with 20 µL of 1.2 N NaOH/0.5 M HEPES. The activated samples were diluted x40 in Reagent Diluent. Diluted samples, controls and standards were added in duplicates to each 96-well plate and incubated for 2 h, followed by incubation with detection antibody for an additional 2 h, with thorough washing between steps. After repeating the plate wash, Streptavidin-HRP B was added and incubated for 20 min. Plates were washed and added substrate solution for 20 min, after which Stop Solution was added. Absorbance was measured immediately at 450 nm with correction at 540 nm. The range of the ELISA assay was 31.3–2,000 pg/mL. Three controls were included on each plate; two serum pools underwent the same steps as the study samples (1,900 pg/mL and 13,000 pg/mL) and one control serum pool was added recombinant TGFβ (R&D Systems) to obtain a concentration of 1,000 pg/mL.

A cut-off was not established by the manufacturer, and there is no consensus cut-off in the literature for this specific kit. Therefore, the cut-off for TGFβ used in the present study was based on receiver operating characteristics (ROC) analysis, and the cut-off was defined as the value resulting in the highest accuracy for predicting recurrence with a balanced grouping. A TGFβ level < 14,943 pg/mL was considered normal.

### Development of a combined biomarker score

A simple cumulative score was developed by adding one for each biomarker with an abnormal value meaning that the score is ranging between 0 (no abnormal values) and 4 (all values abnormal). Due to the cohort size, scores 0–1 were pooled based on visual inspection of a Kaplan Meier graph of RFS, as were the scores 2–4 (Supplementary Figure S1).

### Statistical analysis

Study data were collected and managed using REDCap electronic data capture tools hosted at Open Patient Data Explorative Network and exported to statistical software (Harris et al. 2009, 2019).

Continuous variables were visually inspected for normality using a histogram and a QQ-plot. Normally distributed continuous variables were reported as mean (standard deviation, SD), and continuous variables following a non-normal distribution were reported as median (interquartile range, IQR).

The Wilcoxon signed rank test was used for comparing biomarker levels between sampling times, and the Wilcoxon rank sum test was used for comparing biomarker levels between groups. ROC analysis including area under the ROC curve (AUC) was used to evaluate the predictive performance of individual biomarkers with a 95% confidence interval (CI). The Chi2 test was used to test the association between normal versus abnormal biomarker results and recurrence.

Potential confounders were identified by a directed acyclic graph (DAG, Supplementary Figure S2) to identify the minimal adjustment set of stage and surgery type (DAGitty v3.1, (Textor et al. 2017).

Survival analysis was performed using Kaplan–Meier statistics and Log-rank tests. Median follow-up time was calculated using the reverse Kaplan Meier method for OS data. Unadjusted hazard ratios (HR) were calculated by univariate Cox regression. A multivariate Cox regression model was developed from the biomarker score and the minimal adjustment set. Violation of the proportional hazards assumption was tested on the basis of Schoenfeld residuals and visual inspection of log log plots. The latter suggested that the proportional hazards assumption might be violated for stage and the combined biomarker score. Stage and biomarker score were tested for interaction with time, and as the interactions were not statistically significant, we concluded that they did not violate the proportional hazards assumption. Internal validation of the Cox regression model was performed by bootstrapped resampling with 1,000 iterations.

Two-sided P values < 0.05 were considered statistically significant, no correction for multiple comparisons was performed. All analyses except the DAG were performed using STATA 19BE (StataCorp LLC, Texas, USA).

## Results

### Patient characteristics

Out of 113 patients initially enrolled, 103 patients were included in the statistical analyses. One patient did not have any blood samples collected, two patients were found to be incurable, three patients did not undergo surgery, two patients were converted to radiotherapy and two patients were diagnosed with other cancers. There were 43 patients who had disease recurrence, 37 who died, and the median follow-up was 71 months.

The patients had a mean age of 67.8 years, were evenly divided in terms of sex and the majority had an Eastern Cooperative Oncology Group (ECOG) performance status (PS) of 0 (Table 1). The distribution of histology, stage and smoking status was similar to Danish patients eligible for curative surgery for lung cancer (Christensen et al. 2024; Rasmussen and Jakobsen 2025). The most frequent comorbidity was cardiovascular disease. The p-values in Table 1 are descriptive and were not used for variable selection.

**Table 1:** Patient characteristics. Continuous data are presented as mean (standard deviation), and categorical data are presented as number (percentage). COPD, chronic obstructive pulmonary disease

	No recurrence	Recurrence	Total	*P*-value
*N*	60 (58.3%)	43 (41.7%)	103 (100.0%)	
Age	67.1 (8.8)	68.8 (8.2)	67.8 (8.6)	0.330
Sex, female	32 (53.3%)	20 (46.5%)	52 (50.5%)	0.495
Performance status ≥ 1	12 (20.0%)	7 (16.3%)	19 (18.4%)	0.631
Histology				0.353
Adenocarcinoma	32 (53.3%)	29 (67.4%)	61 (59.2%)	
Squamous cell carcinoma	24 (40.0%)	11 (25.6%)	35 (34.0%)	
Other	4 (6.7%)	3 (7.0%)	7 (6.8%)	
Stage				0.006
1	36 (60.0%)	17 (39.5%)	53 (51.5%)	
2	18 (30.0%)	11 (25.6%)	29 (28.2%)	
3	4 (6.7%)	15 (34.9%)	19 (18.4%)	
4^a^	1 (1.7%)	0 (0.0%)	1 (1.0%)	
Indeterminate^b^	1 (1.7%)	0 (0.0%)	1 (1.0%)	
Comorbidity				
COPD	5 (8.3%)	10 (23.3%)	15 (14.6%)	0.034
Rheumatic disease	10 (16.7%)	1 (2.3%)	11 (10.7%)	0.020
Cardiovascular disease^c^	28 (46.7%)	21 (48.8%)	49 (47.6%)	0.828
Diabetes	5 (8.3%)	3 (7.0%)	8 (7.8%)	0.800
Prior cancer	7 (11.7%)	7 (16.3%)	14 (13.6%)	0.501
Smoking status				0.177
Never	5 (8.3%)	6 (14.0%)	11 (10.7%)	
Former	36 (60.0%)	30 (69.8%)	66 (64.1%)	
Active	19 (31.7%)	7 (16.3%)	26 (25.2%)	
Pack years	38.0 (25.2)	34.0 (17.1)	36.4 (22.2)	0.401

^a^ Patient with a pericardial nodule and a solitary liver metastasis, who received post-operative radio frequency ablation

^b^ Patient with unknown N-stage, who underwent curative surgery

^c^ Including hypertension

### Treatment and complications

The surgical approach was mainly thoracoscopic with lobectomy as the most common procedure, accounting for 87% of cases. Two patients had a pneumonectomy performed. Nearly 60% of procedures were uncomplicated, and the most frequent complication was pneumothorax. There were two cases of 30-day post-operative mortality. A complete overview of treatments and complications is provided in Supplementary Table S1.

### Biomarker dynamics

**Fig. 1:**
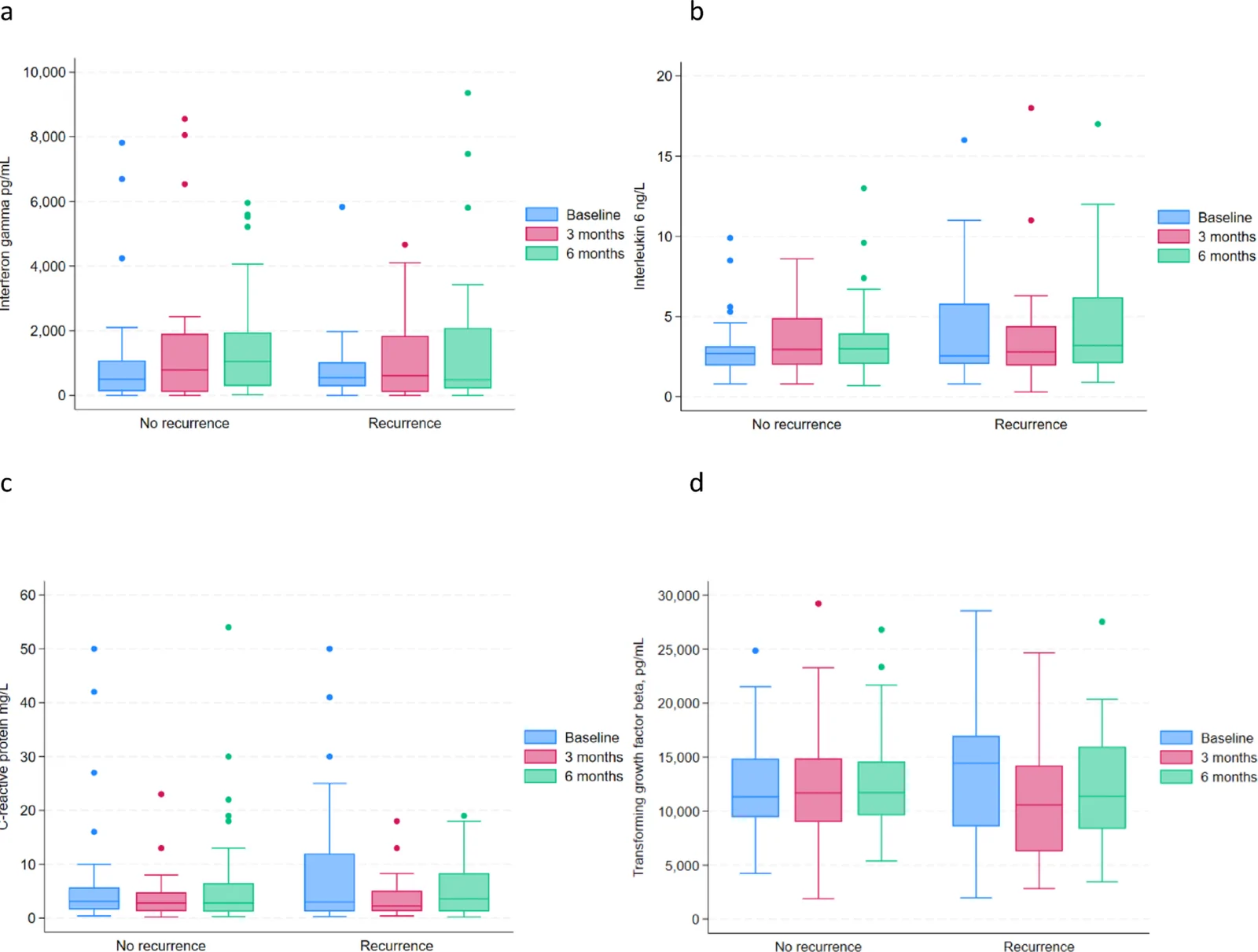
Box plot of (**a**) Interferon gamma as a proxy for Natural Killer cell activity, (**b**) Interleukin 6, (**c**) C-reactive protein, and (**d**) Transforming growth factor beta. Outliers are cropped for better visual overview. The uncropped graphs are available in Supplementary Figure S3. The boxes are grouped according to recurrence status and represented at the three sampling time points (baseline = blue, 3 months = red, 6 months = green)

IFNγ as a proxy for NKA had a baseline median value of 480 pg/mL and 677 pg/mL, respectively, for patients with and without recurrence (Fig. 1a). The concentration remained stable at 467 pg/mL for patients with recurrence (*p* = 0.136), while recurrence free patients experienced an increase to a median of 1,071 pg/mL at 6-months follow-up (*p* = 0.050).

IL6 had a median value at baseline of 2.7 ng/L and 2.8 ng/L, respectively, for patients with and without recurrence (Fig. 1b). There was a non-significant increase to 4.1 ng/L (*p* = 0.086) and 3.3 ng/L (*p* = 0.066), respectively, for the two groups at the 6-months follow-up.

CRP remained relatively stable with baseline median values of 3.2 mg/L and 3.1 mg/L, respectively, for patients with and without recurrence, and 6-months median values of 4.1 mg/L (*p* = 0.488) and 3.0 mg/L (*p* = 0.852, Fig. 1c).

Recurrence free patients maintained stable levels of TGFβ ranging from a median 11,323 pg/mL at baseline to 11,714 pg/mL at the 6-months follow-up (*p* = 0.287, Fig. 1d). Patients with recurrence experienced a non-significant decrease from a median 14,437 pg/mL at baseline to 11,359 pg/mL at the 6-months follow-up (*p* = 0.197).

### Inflammation and immunologic biomarkers as predictors of disease recurrence

Each biomarker was evaluated by ROC analysis in relation to their ability to predict recurrence of NSCLC after curative surgery. NKA at baseline achieved an AUC of 0.55 (95% CI 0.45–0.66), and the cut-off of 250 pg/mL did not predict recurrence with statistical significance (*p* = 0.709, Chi2). Baseline values of IL6 resulted in an AUC of 0.53 (95% CI 0.43–0.63), and the cut-off of 7 ng/L was not associated with recurrence (*p* = 0.305). Similar results were found for CRP with a baseline AUC of 0.52 (95% CI 0.42–0.62) and no significant association with recurrence (*p* = 0.684). TGFβ at baseline had a marginally higher AUC of 0.58 (95% CI 0.48–0.68), and the cut-off determined at 14,943 pg/mL was significantly associated with disease recurrence (*p* = 0.008). An AUC of 0.52–0.58 indicates performance essentially no better than random chance, which is also reflected in the 95% CIs all including 0.5. ROC graphs are available for baseline in Supplementary Figure S4 and at three months follow-up in Supplementary Figure S5.

### Inflammation and immunologic biomarkers at baseline in association with survival

**Fig. 2:**
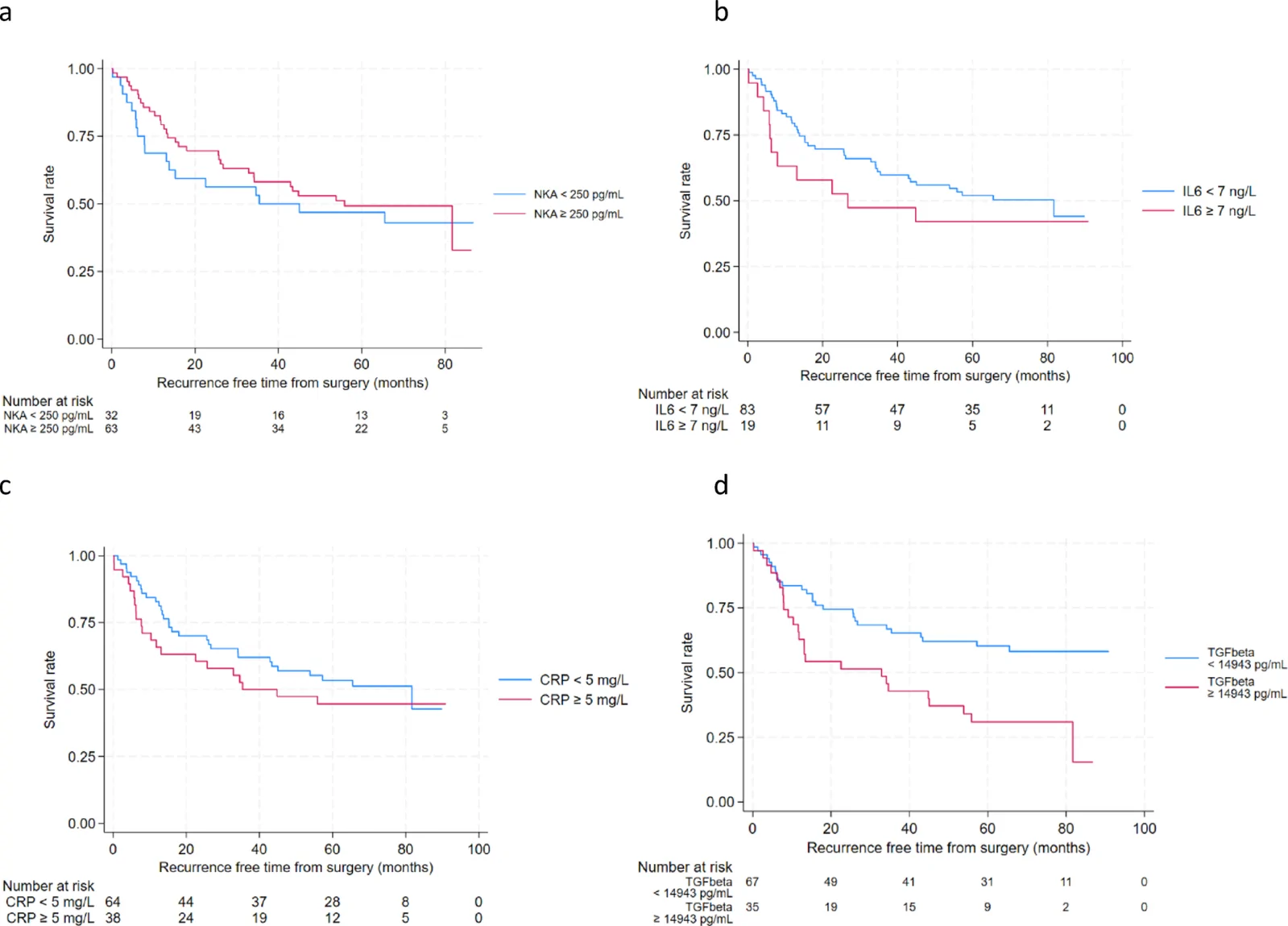
Kaplan-Meier graphs illustrating the association between recurrence free survival and (**a**) Natural Killer cell activity (NKA, *p* = 0.527), (**b**) Interleukin 6 (IL6, *p* = 0.281), (**c**) C-reactive protein (CRP, *p* = 0.367), and (**d**) Transforming growth factor beta (TGFβ, *p* = 0.003)

Each biomarker was evaluated in relation to RFS (Fig. 2). NKA was not significantly associated with RFS with median survival times of 35 and 56 months, respectively, for low NKA and normal NKA (p_log−rank_ = 0.527, Fig. 2a). Patients with IL6 above the cut-off had a shorter median RFS of 27 months vs. 82 months for the patients with a normal level, although the findings were not statistically significant (*p* = 0.281, Fig. 2b). CRP showed similar results with a median RFS of 35 months in the group with elevated CRP vs. 82 months in the group below the cut-off (*p* = 0.367, Fig. 2c). Only baseline TGFβ > 14,943 pg/mL was a statistically significant indicator of poorer RFS (*p* = 0.003, Fig. 2d). The group with abnormal TGFβ had a median RFS of 33 months, while median RFS was not reached for the other group.

**Fig. 3:**
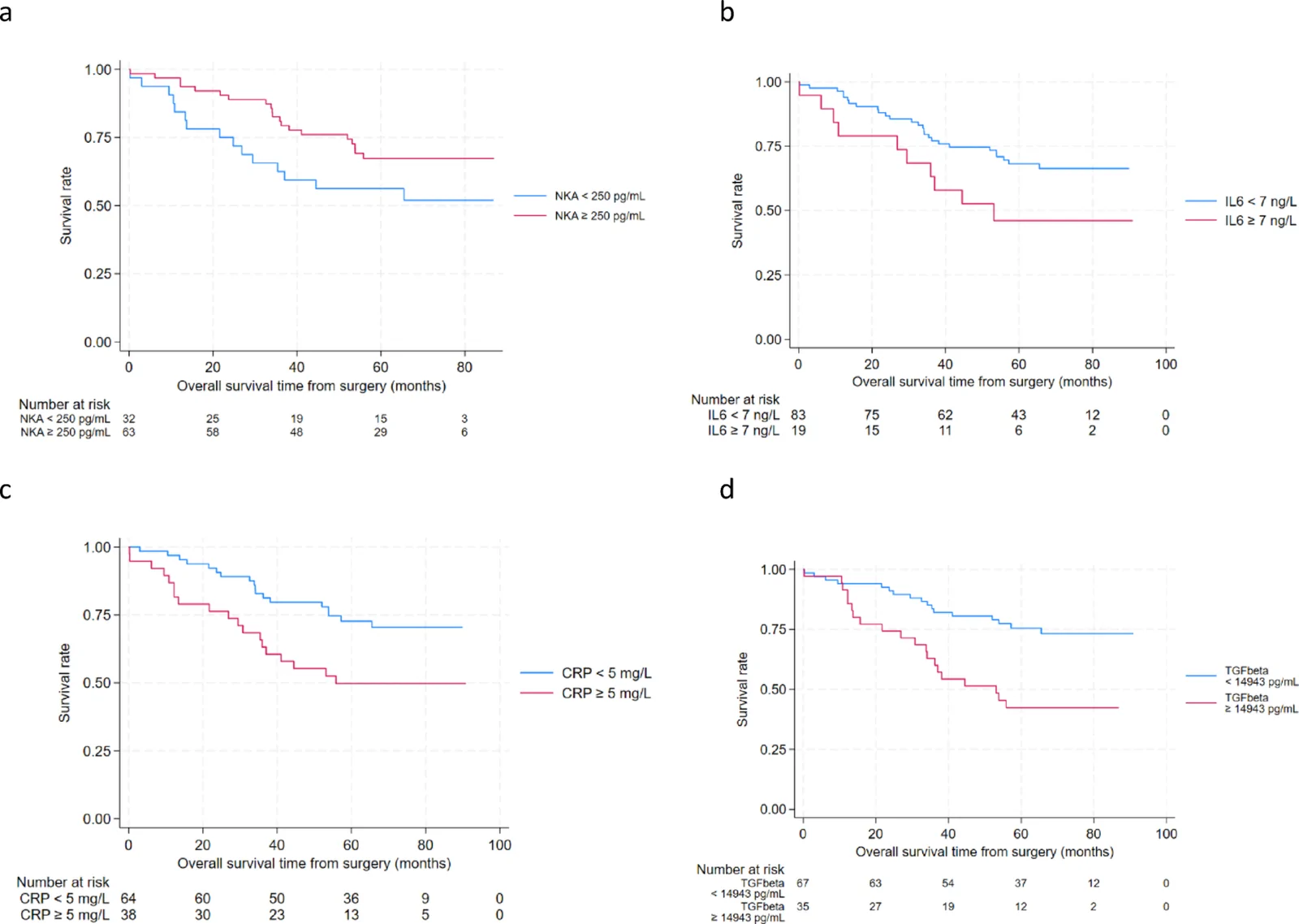
Kaplan-Meier graphs illustrating the association between overall survival and (**a**) Natural Killer cell activity (NKA, *p* = 0.102), (**b**) Interleukin 6 (IL6, *p* = 0.054), (**c**) C-reactive protein (CRP, *p* = 0.015), and (**d**) Transforming growth factor beta (TGFβ, *p* = 0.001)

The prognostic signal was clearer in the OS analyses (Fig. 3). Although there was a visual trend, abnormal NKA (Fig. 3a) and IL6 (Fig. 3b) were not significantly associated with poorer OS (*p* = 0.102 and *p* = 0.054, respectively, for NKA and IL6). Median OS was not reached except for the group with elevated IL6 (53 months). Patients with CRP above the cut-off experienced significantly shorter OS of a median 56 months, which was not reached for the group below the cut-off (*p* = 0.015, Fig. 3c). TGFβ achieved the best separation between the groups (*p* = 0.001, Fig. 3d).

### A biomarker score combining Natural Killer cell activity, Interleukin 6, C-reactive protein and Transforming growth factor beta at baseline

**Fig. 4:**
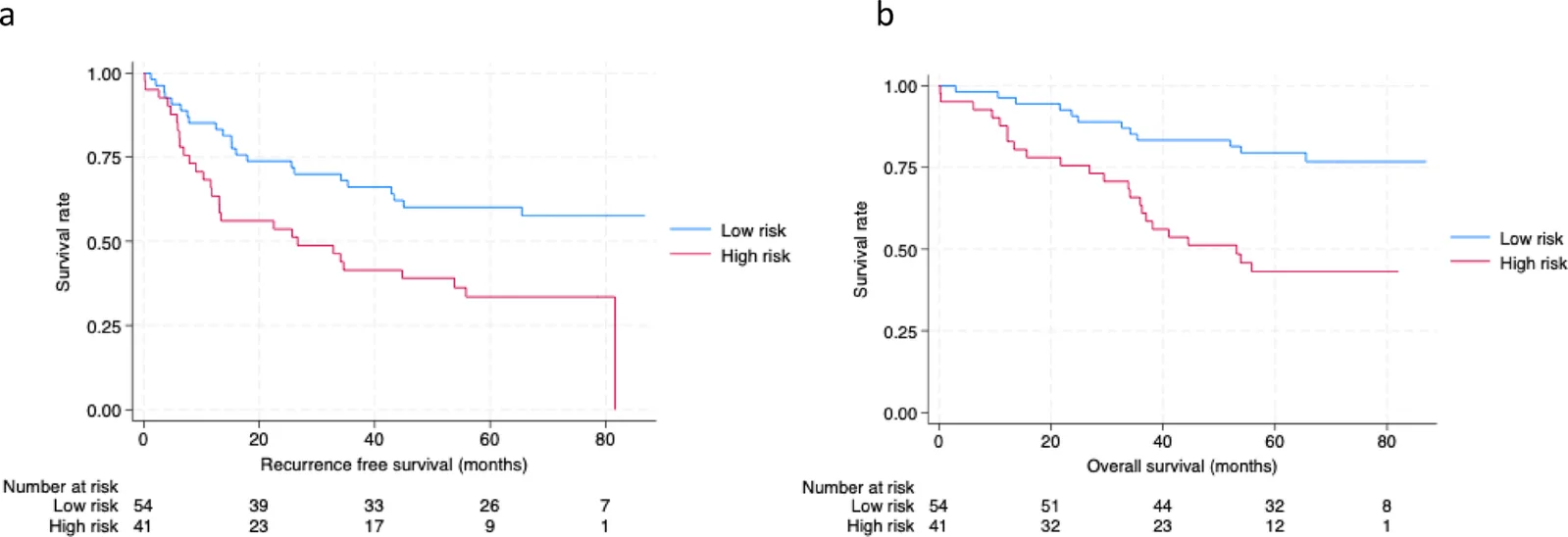
Kaplan-Meier graphs depicting the two-category score in relation to (**a**) recurrence free survival (*p* = 0.006) and (**b**) overall survival (*p* < 0.001)

 The score was a simple cumulative system from 0 to 4 with 0 equalling no abnormal biomarker results and 4 equalling all abnormal biomarker results. The patients with scores 0–1 and 2–4 were termed low risk and high risk, respectively (Fig. 4). These groups were strongly associated with both RFS (*p* = 0.006, Fig. 4a) and OS (*p* < 0.001, Fig. 4b).

A high-risk biomarker score resulted in an unadjusted HR of 2.17 (95% CI 1.24–3.81, *p* = 0.007) for RFS and an unadjusted HR of 3.28 (95% CI 1.62–6.61, *p* = 0.001) for OS when compared to the low-risk group. The adjusted HR was 1.93 (95% CI 1.06–3.51, *p* = 0.032) for RFS and 3.18 (95% CI 1.53–6.61, *p* = 0.002) for OS, when the minimal adjustment set was accounted for. The DAG and the minimal adjustment set consisting of stage and surgery type are described in more detail in the Supplementary Information Figure S2.

Internal validation using bootstrap resampling with 1,000 iterations produced similar results with an adjusted HR of 3.18 (95% CI 1.49–6.75, *p* = 0.003) for the high-risk biomarker score compared to low-risk in relation to OS. Sensitivity analyses excluding TGFβ from the biomarker score and evaluating an alternative adjustment set of clinical variables as well as incorporating the biomarker score as a continuous variable supported the direction of the biomarker score (Supplementary Information Table S2–S7). The biomarker score remained significantly associated with OS in the alternative adjustment set and when modeled as a continuous variable, while the score did not retain statistical significance as a continuous marker for RFS.

## Discussion

Curative surgery offers the best prognosis for eligible patients with NSCLC, and the patient’s inflammation grade and immunologic status may affect their survival. In this hypothesis generating study, we found that, individually, NKA, IL6, CRP and TGFβ were not suitable markers for predicting recurrence, but abnormal levels were associated with poorer RFS and OS when condensed into a biomarker score.

CRP and IL6 are widely established as markers of inflammation, and their association with prognosis in patients with lung cancer is well-known (Allin and Nordestgaard 2011; Liu et al. 2022; Han et al. 2024). We also found a statistically significant association between increased CRP and OS confirming previous reports, but this secondary finding should be interpreted with caution as the results were not corrected for multiple testing. The ability of CRP and IL6 to predict disease recurrence in patients treated with curative intent has been suggested but is not thoroughly investigated (Kita et al. 2011; Watanabe et al. 2021), and our data suggest that CRP and IL6 do not have a role in recurrence detection in the present set-up. NKA has previously shown both diagnostic and prognostic potential in patients with NSCLC (Choi et al. 2019; Wen et al. 2023). We therefore hypothesized that the status of the innate immune system as indicated by NKA might be associated with disease recurrence. TGFβ can affect the function and possibly the activity of NK cells. TGFβ did achieve the highest AUC, though only marginally better than chance. An elevated level of TGFβ was, as the only one of the four biomarkers, associated with recurrence. It was, however, also the only biomarker with a data driven cut-off, which may explain the superior performance compared to NKA, CRP and IL6.

It is well established that NK cells play a crucial role in eliminating malignant cells, and it is reasonable to hypothesise that NKA may hold prognostic value in lung cancer, which has been demonstrated in other cancer types (Hansen et al. 2019). NK cells control tumours not only through their cytotoxic effect but also via regulatory functions as they secrete cytokines, e.g. IFNγ, which have direct anti-tumour effects and can recruit immune cells to the tumour site to mount a coordinated immune response (Long et al. 2013; Jiang and Jiang 2023). Tumours can evade surveillance by inducing local and systemic inhibition of NK cell function. We investigated whether the regulatory activity of NK cells in peripheral blood has prognostic significance and IFNγ levels were measured as a proxy for NKA, acknowledging the functional distinction between cytotoxic and regulatory NK subsets. In a previous study using the same analytical platform, early-stage patients exhibited NKA levels comparable to non-cancer controls (Borg et al. 2022). In the present study, recurrence-free patients showed higher NKA levels at six months, although barely reaching statistical significance. These findings suggest that stage-specific cut-off values may be warranted for a more accurate assessment of NKA as a prognostic marker.

We found that combining the four biomarkers into a score was more informative and was better at stratifying the patients into significantly different prognostic groups. All biomarkers were assigned equal weight in the biomarker score. This was because it is simple and easy to understand, and we have no solid evidence for which weight to assign to each of the four biomarkers. We might also risk wrongfully assigning too much weight to TGFβ, since the cut-off is data-driven. The cut-off between low risk and high risk at ≥ 2 abnormal biomarker results was based on visual inspection of the Kaplan Meier graphs and clinical reasoning. We hypothesized that patients with 0 or 1 abnormal biomarker result would have a better prognosis than patients with multiple abnormal biomarker results. When adjusted for the critical potential confounders in the minimal adjustment set, the biomarker score remained an independent prognostic factor for OS.

The concept of a biomarker panel or score is widely used because it often improves sensitivity and may therefore amplify the observed signal. Lee et al. combined NKA, carcinoembryonic antigen (CEA) and cancer antigen (CA) 19 − 9 and found that the combined panel had superior performance compared to individual biomarkers for detection of gastric cancer (Lee et al. 2017). Kjaergaard et al. investigated a combination of CRP, CA 19 − 9, IL6 and YKL-40 for predicting one-year mortality in a cohort of pancreatic cancer patients and found a six-fold increase for the patients with the highest compared to the lowest biomarker score (Kjaergaard et al. 2021). The four biomarkers in the present study represent non-specific inflammation in combination with host immunology, which are two aspects of the multifactorial process of tumourigenesis. An ideal biomarker approach may combine multiple hallmarks of cancer in order to cover as many changes as possible. The prognostic impact of the present biomarker score may benefit from combination with tumour specific biomarkers such as circulating tumour DNA, effectors from the tumour microenvironment such as angiogenic factors or extracellular matrix signalling components.

The present study sought to employ simple analysis methods for easier future implementation. Therefore, CRP and IL6 were measured on the COBAS system using the same kits as for the routine blood analyses used in local clinical practice. CRP may also have been analysed as high-sensitivity CRP (Muller et al. 2019), which could have improved the detection, but it is rarely used in routine clinical practice in Denmark and has a higher cost. Similarly, IL6 can also be analysed by an ELISA, which is more labour-intensive, or on the Simoa^®^ System, which may have a larger detection range, but has a higher price-point (Lasseter et al. 2020). Most often NKA is reported as the ability of NK cells to perform cytotoxicity measured either by the ^51^chromium release assay (Elsner and Dressel 2020) or by more recent flow cytometry-based assays (Valiathan et al. 2012). However, these approaches are labour-intensive, and the ^51^chromium release assay requires handling and disposal of radioactive material. The NK Vue^®^ test quantifies the regulatory ability of these cells as an alternative measure of NKA. This approach offers key advantages including simplicity and minimal hands-on time compared to many other methods, making it well suited for routine clinical use. TGFβ was analysed by a commercially available ELISA kit, and the method may preferentially be set up on another system for ease and scalability in the future.

The main study limitation was the size of the cohort. With around 100 patients and a recurrence rate just above 40%, the study may risk type 2 errors due to lack of statistical power. A case-control design would offer us a larger cohort and allow us to balance the groups according to recurrence, but the prospective observational design with consecutively enrolled patients confers the benefit of a more realistic setting. However, the patient recruitment process was long resulting in substantially different follow-up durations. This may have affected the results, because lung cancer treatment continues to improve which could potentially increase OS for patients more recently recruited. However, a sensitivity analysis including recruitment period (binary; first half vs. second half) as a variable in the multiple Cox regression analysis pointed towards higher HR for patients recruited in the second half of the period though this was not statistically significant. The study did not include a control group of healthy subjects or patients with non-malignant diseases but relied on established cut-offs for the biomarkers except for TGFβ. The data driven cut-off for TGFβ is a considerable caveat in the interpretation of our results, as it may have artificially inflated the observed effect sizes. There is a substantial risk of overfitting because the cut-off has not been validated in an external cohort, and therefore the generalisability of the results is limited. No final conclusions can be drawn until TGFβ – and the biomarker score – is validated in an independent study. A validation study is planned using samples from an independent cohort of patients currently being recruited at Lillebaelt Hospital Vejle. We had no information about lung function or immune modulating medication, which may have impacted the results. The samples were analysed after a storage time ranging from 48 to 91 months with the exception of NKA, which was analysed within one month of sampling. The prolonged storage time hypothetically may have affected the biomarker results (Enroth et al. 2016). However, very few studies have addressed this issue and we are not aware of any studies utilising the same platforms and kits as the present study making direct comparison difficult. The differential storage time of the NKA samples compared to the other samples may have affected the biomarker results, but all biomarkers of the same type were analysed within the same time range limiting the potential bias. Pre-analytical handling was done according to laboratory standard operating procedures by experienced laboratory technicians ensuring a uniformly high quality.

## Conclusion

We found that, individually, NKA, CRP, IL6 and TGFβ were not suitable for predicting disease recurrence after curative surgery for lung cancer. When combined in a binary risk score, the biomarker panel was significantly associated with OS and was an independent prognostic factor. The combined biomarker score may be used in future studies to give an indication of the inflammatory and immunologic status of the patient. It could be useful for pre- or post-surgery assessment, in relation to immunotherapy or other oncologic treatments if clinical utility can be proven.

## Supplementary Information

Below is the link to the electronic supplementary material.

Supplementary Material 1

## Data Availability

Data is not publicly available because they contain sensitive information. Data can be made available upon reasonable request.
