# Supplementary material for: The prognostic potential of an immunology score in patients treated with curative surgery for non-small cell lung cancer
Source: J Cancer Res Clin Oncol. 2026 Jul 21;152(10):195. doi: 10.1007/s00432-026-06562-1 (PMC13672274; doi:10.1007/s00432-026-06562-1)
Supplement: Supplementary file 1 [file 432_2026_6562_MOESM1_ESM.pdf]

Supplementary Information

Title: The prognostic potential of an immunology score in patients treated with curative surgery for non-small cell lung cancer

Journal: Journal of Cancer Research and Clinical Oncology

Sara Witting Christensen Wen[a,b,*], Signe Timm[b,c], Morten Hornemann Borg[b,d], Ole Hilberg[b,d], Torben Frøstrup Hansen[b,c], Christa Haugaard Nyhus[c], Torben Schjødt Hansen[c], Line Nederby[a,b]

a. Department of Biochemistry and Immunology, Lillebaelt Hospital Vejle, University Hospital of Southern Denmark, Beriderbakken 4, 7100 Vejle, Denmark

   sara.witting.christensen.wen@rsyd.dk; ORCID 0000-0003-3755-0922

   line.nederby@rsyd.dk; ORCID 0000-0002-0101-328X

b. Department of Regional Health Research, Faculty of Health Sciences, University of Southern Denmark, Campusvej 55, 5230 Odense, Denmark

c. Department of Oncology, Lillebaelt Hospital Vejle, University Hospital of Southern Denmark, Beriderbakken 4, 7100 Vejle, Denmark

   Signe.timm@rsyd.dk; ORCID 0000-0002-5862-7784

   Torben.hansen@rsyd.dk; ORCID 0000-0001-7476-671X

   christa.haugaard.nyhus@rsyd.dk

   Torben.schjoedt.hansen@rsyd.dk

d. Department of Medicine, Lillebaelt Hospital Vejle, University Hospital of Southern Denmark, Beriderbakken 4, 7100 Vejle, Denmark

   morten.hornemann.borg@rsyd.dk; ORCID 0000-0002-6204-2623

   ole.hilberg@rsyd.dk; ORCID 0000-0002-3075-3463

*Corresponding author

a

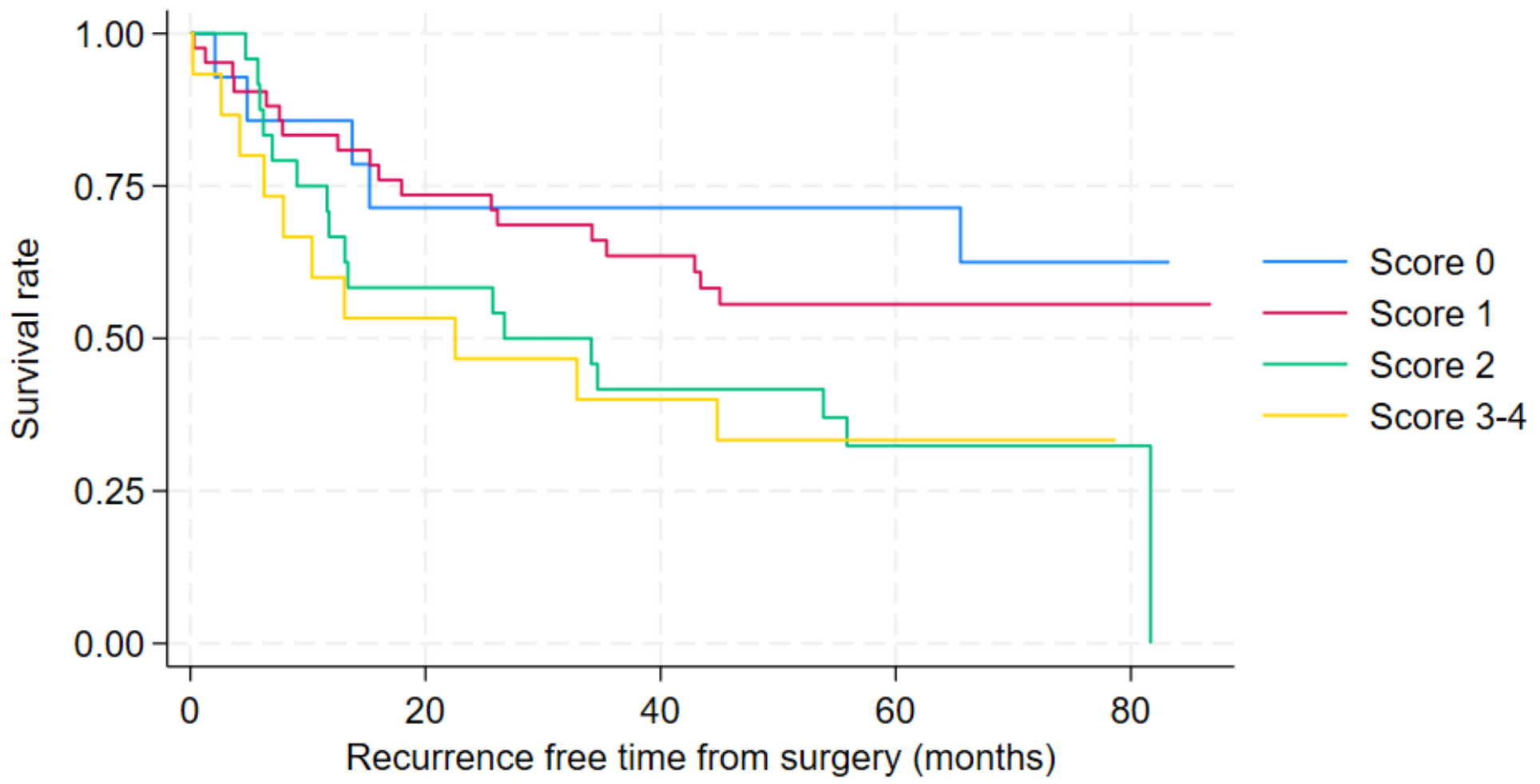

Number at risk					
Score 0	14	10	10	9	2
Score 1	42	30	24	18	5
Score 2	24	14	10	5	1
Score 3-4	15	8	6	3	0

b

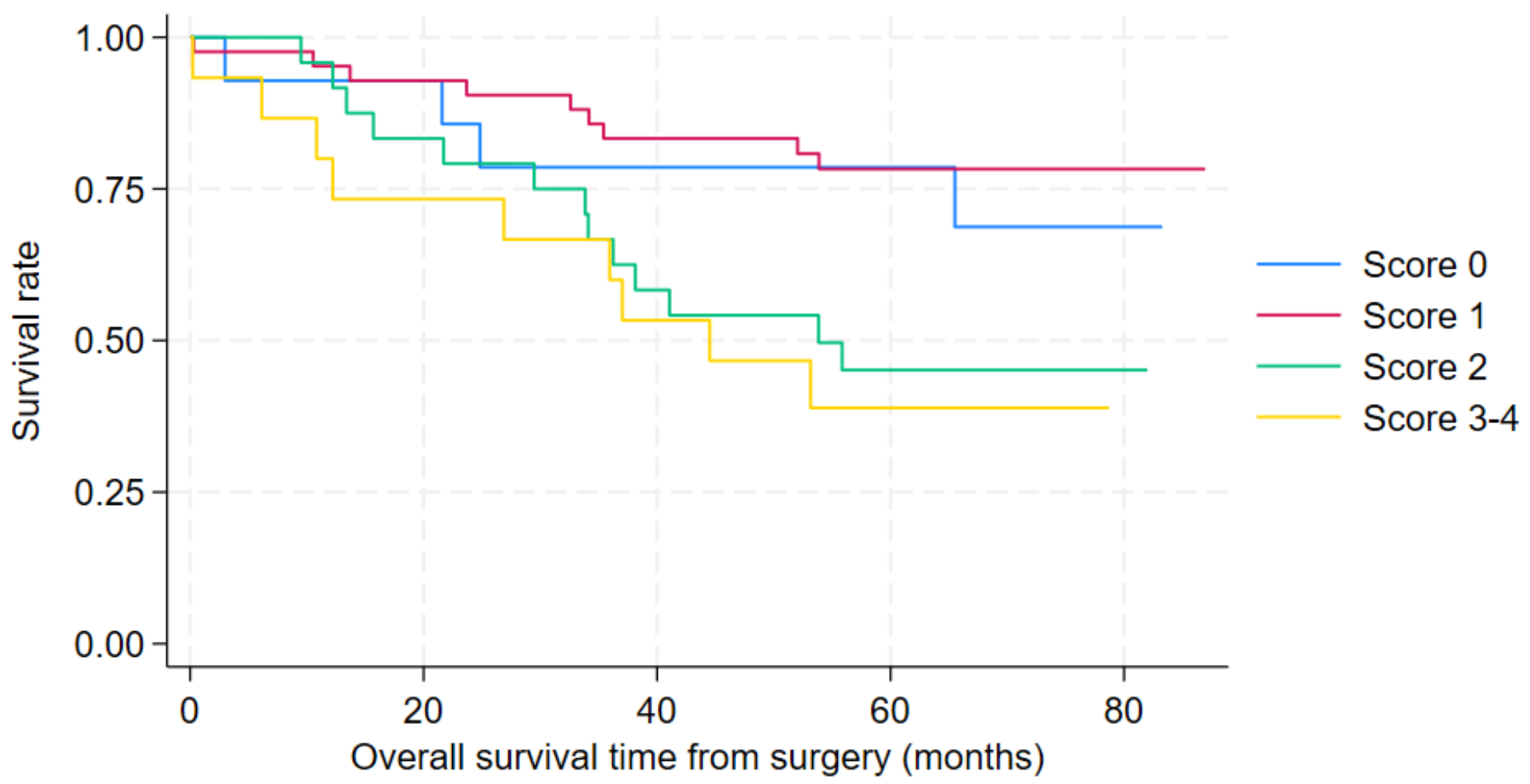

Number at risk					
Score 0	14	13	11	9	2
Score 1	42	39	34	24	6
Score 2	24	20	14	8	1
Score 3-4	15	11	8	3	0

Fig. S1 Kaplan Meier graphs illustrating the association between the four-tier biomarker score and (a) Recurrence free survival (p = 0.0530, Log rank) and (b) Overall survival (p = 0.0076).

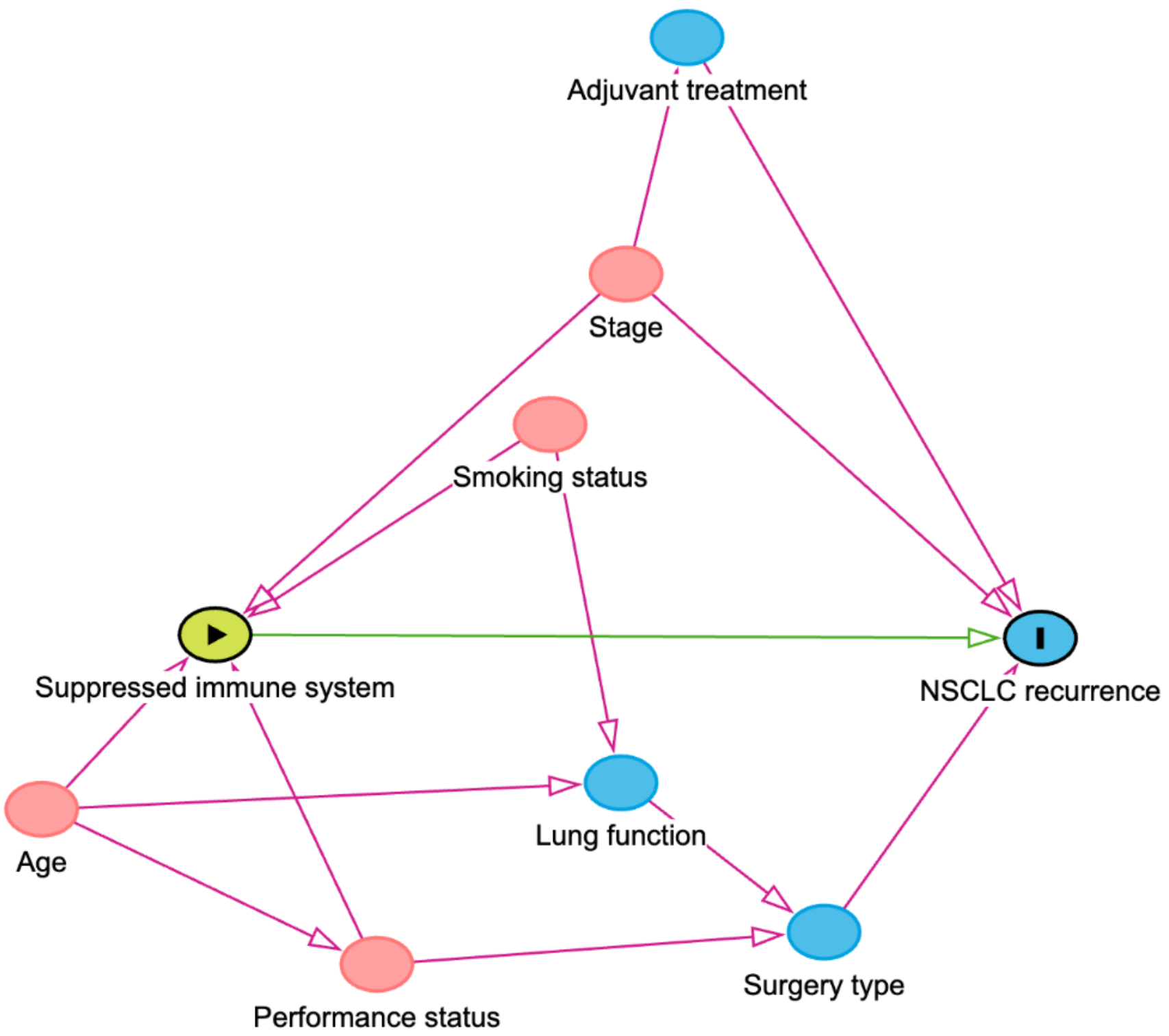

Fig. S2 Directed acyclic graph for establishing the minimal adjustment set of covariates for multiple regression analyses.

Minimal adjustment set, three options:

1: Age, Performance status, Smoking status, Stage

2: Lung function, Performance status, Stage

3: Stage, Surgery type

	No recurrence	Recurrence	Total
N	60 (58.3%)	43 (41.7%)	103 (100.0%)
Laparoscopic			
No	14 (23.7%)	14 (35.0%)	28 (28.3%)
Yes	45 (76.3%)	26 (65.0%)	71 (71.7%)
Surgery type			
Lobectomy	53 (88.3%)	37 (86.0%)	90 (87.4%)
Pulmonectomy	0 (0.0%)	2 (4.7%)	2 (1.9%)
Other	7 (11.7%)	4 (9.3%)	11 (10.7%)
Surgery complications			
No	41 (68.3%)	19 (46.3%)	60 (59.4%)
Pneumothorax	12 (20.0%)	10 (23.3%)	22 (21.4%)
Infection	3 (5.0%)	3 (7.0%)	6 (5.8%)
Bleeding	1 (1.7%)	1 (2.3%)	2 (1.9%)
Death within 30 days	2 (3.3%)	0 (0.0%)	2 (1.9%)
Radical			
No	4 (6.7%)	2 (4.8%)	6 (5.9%)
Yes	55 (91.7%)	39 (92.9%)	94 (92.2%)
Unknown*	1 (1.7%)	1 (2.4%)	2 (2.0%)
Adjuvant treatment			
No	43 (71.7%)	24 (55.8%)	67 (65.0%)
Yes	17 (28.3%)	19 (44.2%)	36 (35.0%)

Table S1 Treatment and complications. Categorical data are presented as number (percentage).

* Two patients had inconclusive assessments for radicality.

a

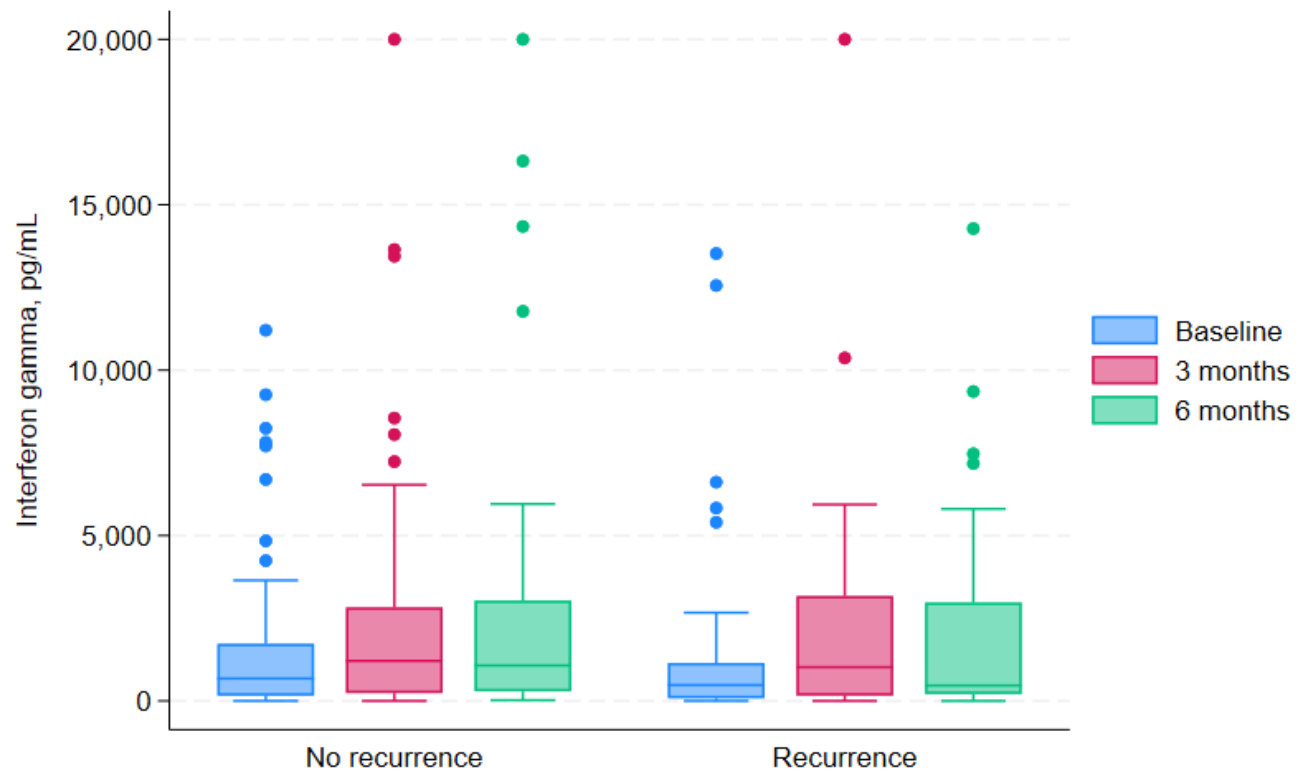

b

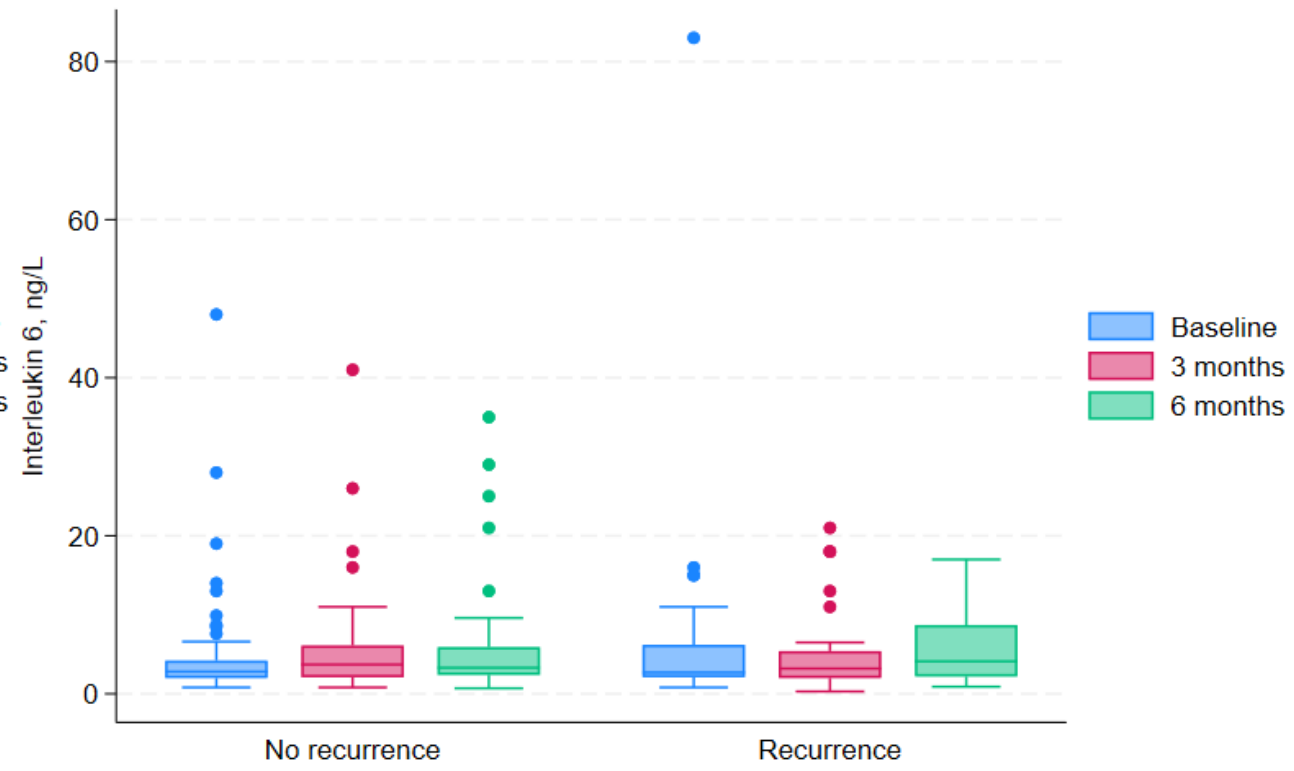

c

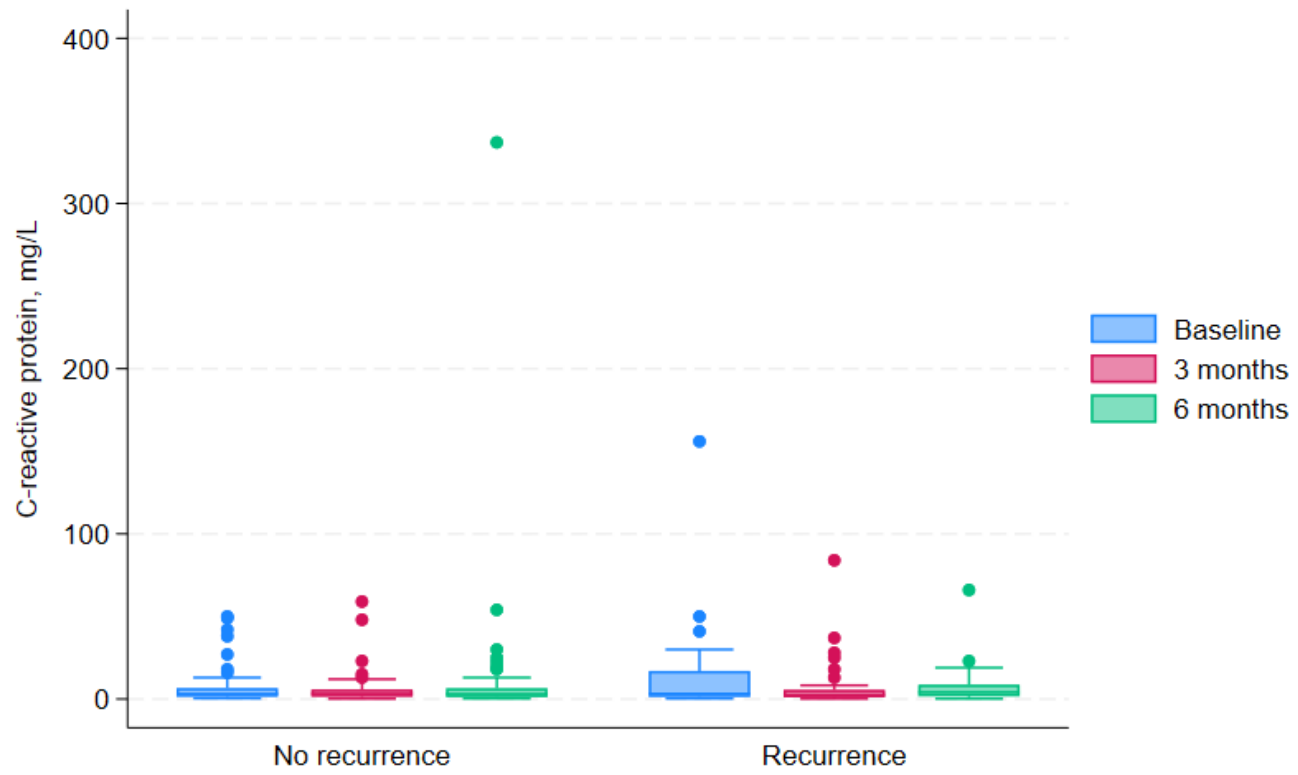

d

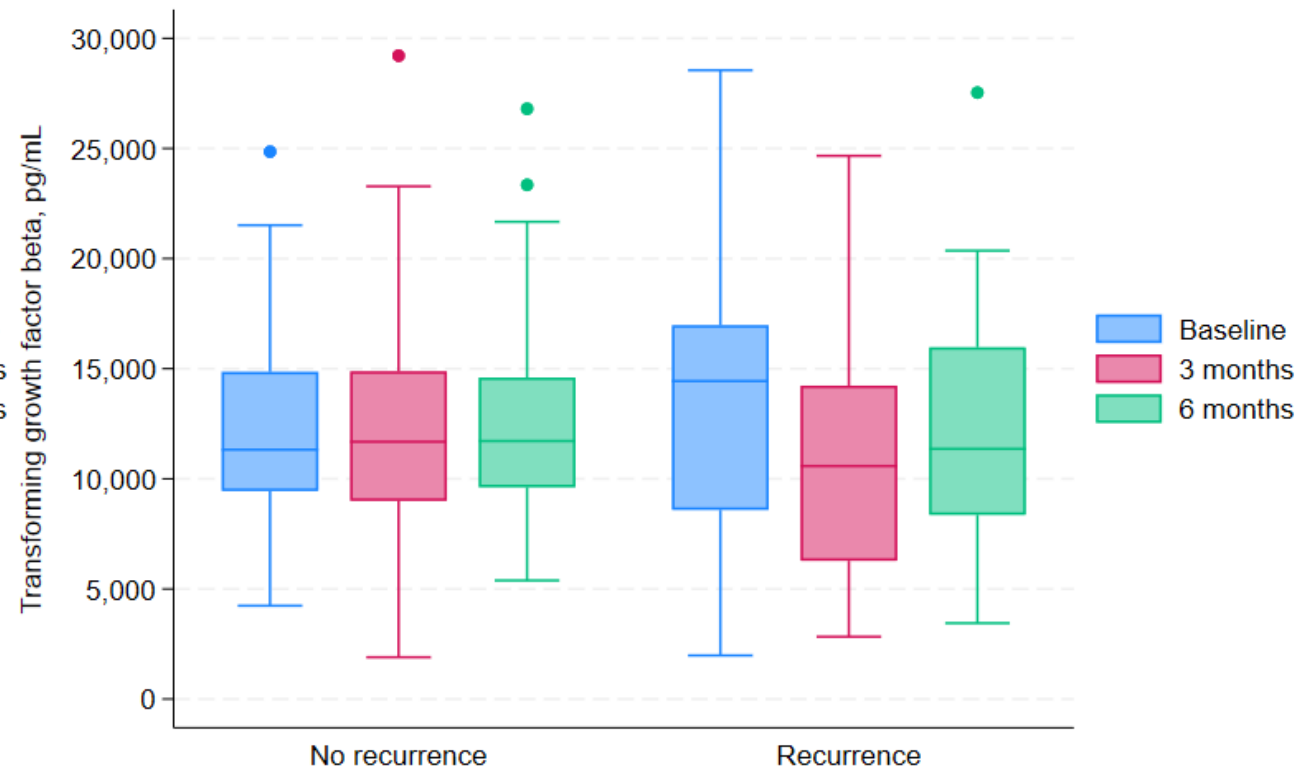

Fig. S3 Box plot of (a) Interferon gamma as a proxy for Natural Killer cell activity, (b) Interleukin 6, (c) C-reactive protein, and (d) Transforming growth factor beta. The boxes are grouped according to recurrence status and represented at the three sampling time points (baseline = blue, 3 months = red, 6 months = green).

a

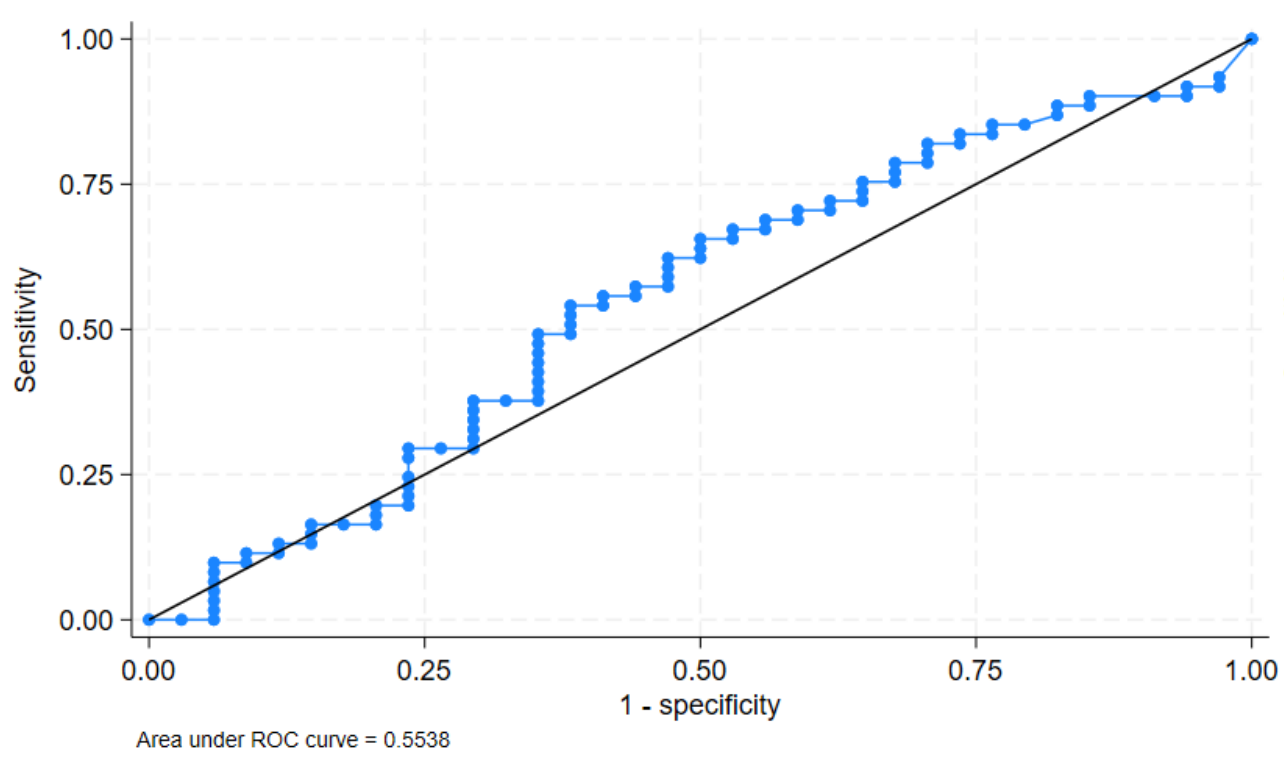

b

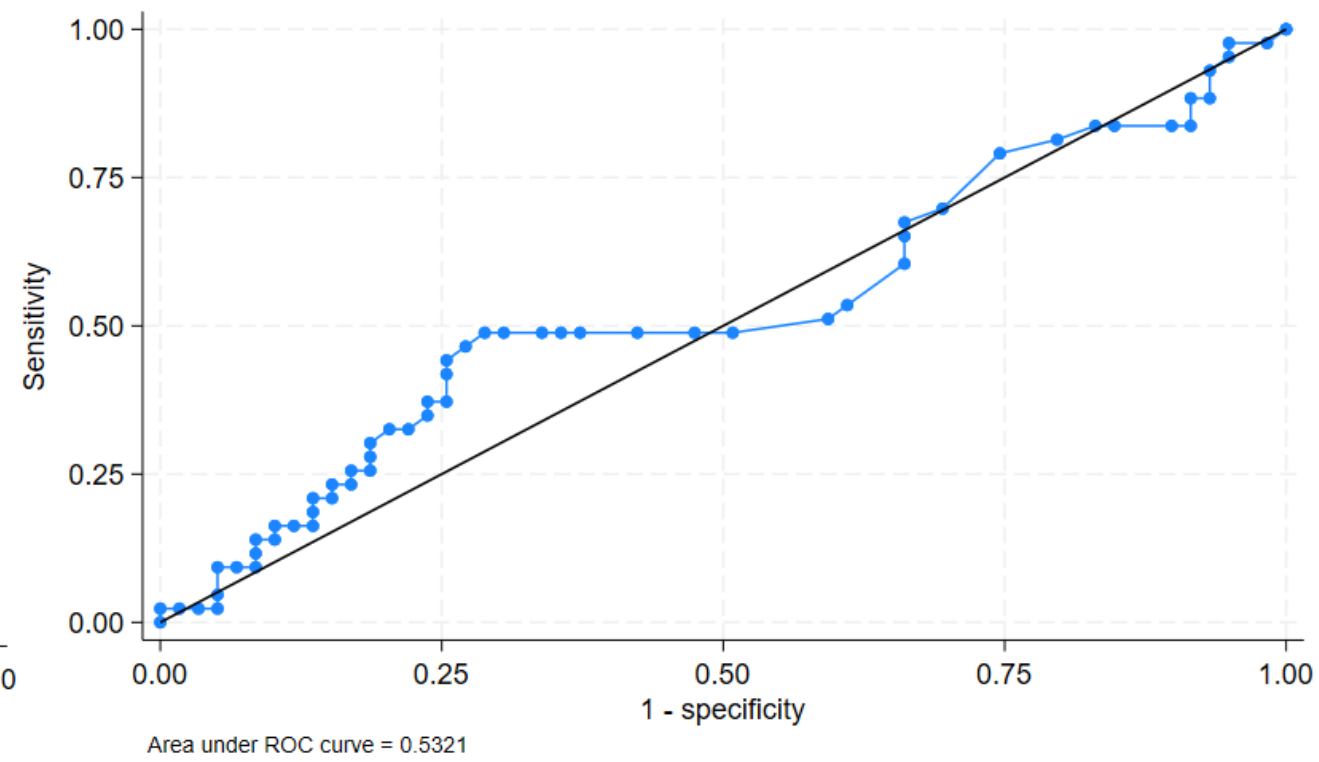

c

d

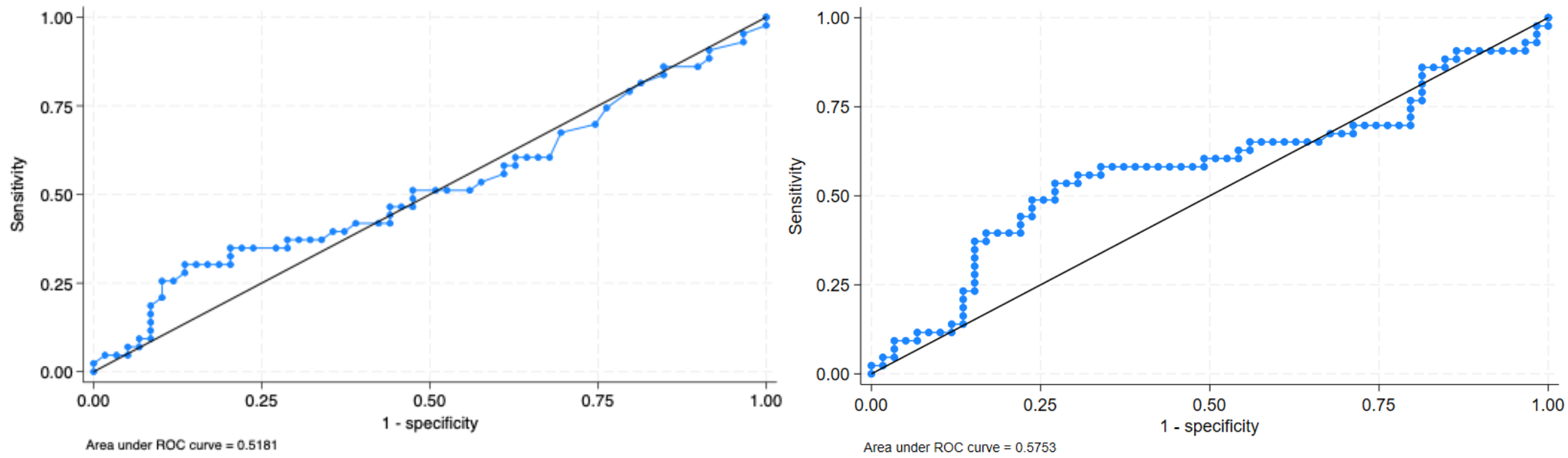

Fig. S4 Receiver operating characteristics (ROC) analysis of (a) Natural Killer cell activity, (b) Interleukin 6, (c) C-reactive protein, and (d) Transforming growth factor beta pre-surgery for predicting recurrence of non-small cell lung cancer after curative surgery.

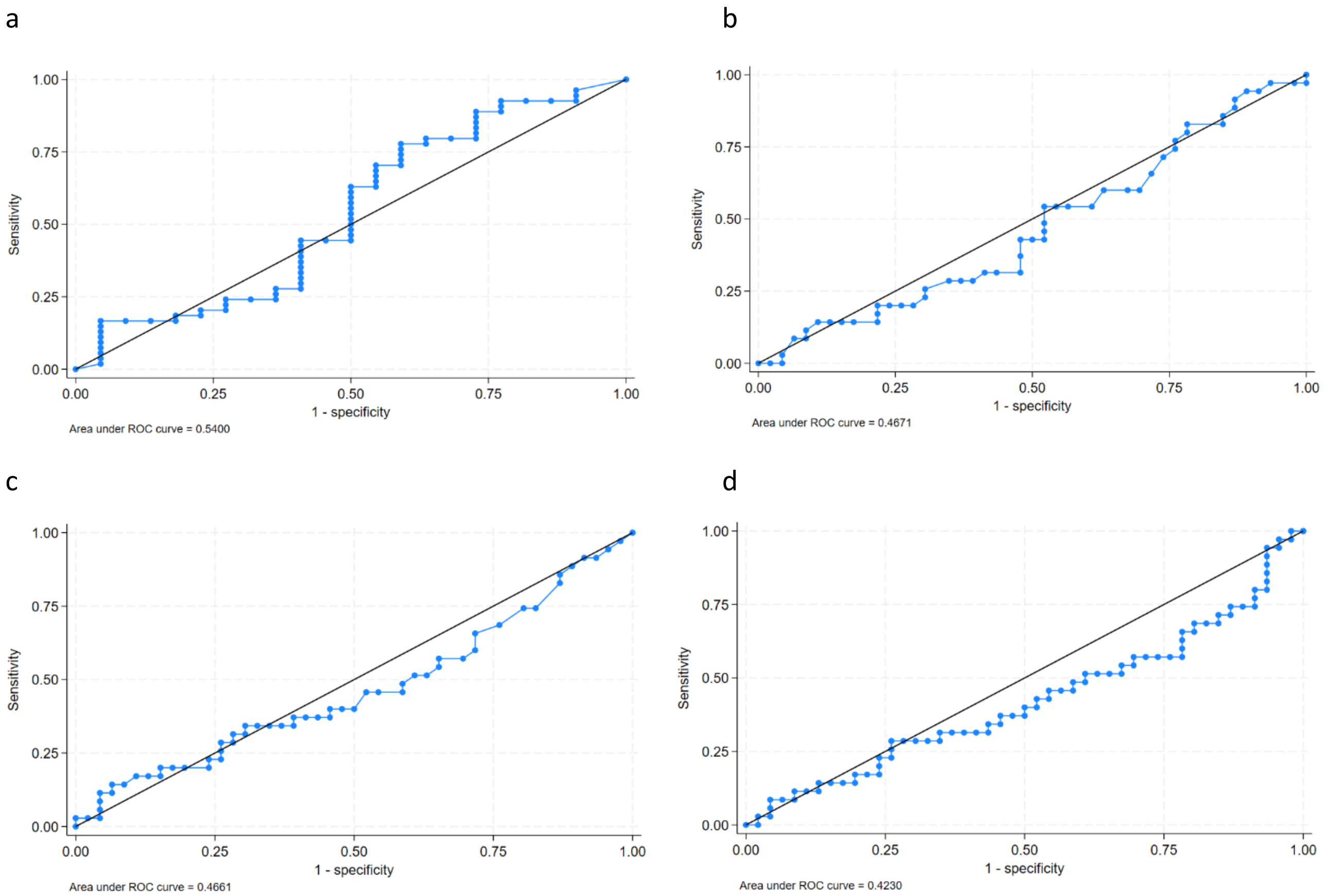

Fig. S5 Receiver operating characteristics (ROC) analysis of (a) Natural Killer cell activity, (b) Interleukin 6, (c) C-reactive protein, and (d) Transforming growth factor beta at three months of follow-up for predicting recurrence of non-small cell lung cancer after curative surgery.

Sensitivity analyses

We constructed a biomarker score in the same way as reported in the manuscript with equal weights but excluding TGFβ. The cut-off was ≥ 2 abnormal results equaling an abnormal test as with the 4-marker score. The 3-marker score resulted in a similar prognostic impact in the cohort.

The 3-marker score did not have a statistically significant association with RFS using neither minimal adjustment set 1 nor 3. The 3-marker score was significantly associated with OS using both minimal adjustment sets. The HRs were 2.4 and 2.5 for the two adjustment sets, respectively, which was somewhat similar to the 4-marker score, which had HRs of around 3.1 in both adjustment sets. Harrell's C for the 3-marker score without TGFβ = 0.6512 (95% CI 0.56-0.74). Harrell's C for the 4-marker score = 0.6635 (95% CI 0.58-0.75).

Variable	HR	95% CI	P-value
Stage			
≥ 2	1.61	0.84-3.08	0.150
Surgery type			
Other than lobectomy	1.01	0.40-2.53	0.985
Biomarker score			
1	1.33	0.49-3.62	0.579
2	2.37	0.87-6.47	0.093
3-4	2.19	0.72-6.65	0.167

Table S2. Multivariate Cox regression model, RFS, biomarker score ordinal.

Variable	HR	95% CI	P-value
Stage			
≥ 2	1.17	0.55-2.47	0.679
Surgery type			
Other than lobectomy	1.27	0.42-3.83	0.677
Biomarker score			
1	0.70	0.21-2.30	0.558
2	2.17	0.70-674	0.180
3-4	2.56	0.75-8.77	0.135

Table S3. Multivariate Cox regression model, OS, biomarker score ordinal.

Variable	HR	95% CI	P-value
Stage			
≥ 2	1.59	0.84-3.02	0.156
Surgery type			
Other than lobectomy	0.95	0.39-2.32	0.913
Biomarker score	1.35	0.98-1.85	0.065

Table S4. Multivariate Cox regression model, RFS, biomarker score continuous.

Variable	HR	95% CI	P-value
Stage			

≥ 2	1.22	0.58-2.58	0.598
Surgery type			
Other than lobectomy	1.05	0.35-3.11	0.930
Biomarker score	1.64	1.11-2.41	0.012

Table S5. Multivariate Cox regression model, OS, biomarker score continuous.

Variable	HR	95% CI	P-value
Age	1.05	1.01-1.10	0.022
Performance status			
≥ 1	1.52	0.63-3.62	0.350
Smoking status			
Former	0.41	0.14-1.18	0.099
Current	0.34	0.10-1.18	0.088
Stage			
≥ 2	1.44	0.69-3.01	0.326
Biomarker score			
High-risk	3.12	1.42-6.84	0.005

Table S6. Multivariate Cox regression model, OS, minimal adjustment set 1.

Variable	HR	95% CI	P-value
Stage			
≥ 2	1.23	0.60-2.52	0.577
Surgery type			
Other than lokectomy	1.25	0.42-3.75	0.692
Biomarker score			
High-risk	3.18	1.53-6.61	0.002

Table S7. Multivariate Cox regression model, OS, minimal adjustment set 3.
